# Loss of Nef-mediated CD3 down-regulation in the HIV-1 lineage increases viral infectivity and spread

**DOI:** 10.1073/pnas.1921135117

**Published:** 2020-03-16

**Authors:** Dejan Mesner, Dominik Hotter, Frank Kirchhoff, Clare Jolly

**Affiliations:** ^a^Division of Infection and Immunity, University College London, London WC1E 6BT, United Kingdom;; ^b^Institute of Molecular Virology, Ulm University Medical Center, 89081 Ulm, Germany

**Keywords:** HIV, CD3, Nef, T cell, Env

## Abstract

Nef is an accessory protein of primate lentiviruses that is essential for efficient replication and pathogenesis of HIV-1. A conserved feature of Nef proteins from different lentiviral lineages is the ability to modulate host protein trafficking and down-regulate a number of cell surface receptors to enhance replication and promote immune evasion. Notably, the inability of Nef to down-regulate CD3 from infected T cells distinguishes HIV-1 Nef and its direct simian precursors from other primate lentiviruses. Why HIV-1 does not employ this potential immune evasion strategy is not fully understood. Using chimeric HIV-1 constructs expressing lentiviral Nef proteins that differ in their ability to down-modulate CD3, we show that retaining CD3 on the surface of infected primary T cells results in increased viral replication and cell-to-cell spread. We identified increased expression of envelope (Env) trimers at the cell surface and increased Env incorporation into virions as the determinants for the Nef- and CD3-dependent enhancement of viral infectivity. Importantly, this was independent of Nef-mediated antagonism of the host restriction factor SERINC5. CD3 retention on the surface of infected primary T cells also correlated with increased T cell signaling, activation, and cell death during cell-to-cell spread. Taken together, our results show that loss of an otherwise conserved function of Nef has a positive effect on HIV-1 replication, allowing for more efficient replication while potentially contributing to HIV-1 pathogenesis by triggering T cell activation and cell death during viral spread.

## Significance

Lentiviruses encode accessory proteins to manipulate their host cells in order to efficiently replicate and evade antiviral defenses. Interestingly, most lentiviral Nefs down-regulate CD3 from the surface of infected T cells to perturb immune responses. However, for reasons that are incompletely understood, HIV-1 and its simian immunodeficiency virus ancestors lack this function. Here, we report that engineering HIV-1 for Nef-mediated down-regulation of CD3 reduces Env-dependent HIV-1 infectivity, resulting in less efficient cell-to-cell spread and replication. Our data suggest that HIV-1 may have evolved to lose the CD3 down-modulation function of Nef in order to allow T cell activation and to boost viral replication, possibly at the cost of less effective immune evasion and increased pathogenicity.

Natural lentiviral infections of nonhuman primates are usually not associated with viral pathogenesis (1). One well-characterized example of this is simian immunodeficiency virus SIVsmm infection of sooty mangabeys, which show no loss of CD4+ T cells and no progression to AIDS despite high viral loads (reviewed in ref. 1). By contrast, interspecies transmissions of lentiviruses into nonnatural hosts (either rare natural zoonoses or experimental challenge) may cause profoundly pathogenic infection. Such zoonosis is most famously evidenced by HIV-1 that resulted from at least four independent transmissions of SIVcpz and SIVgor into humans, with HIV-1 group M derived from SIVcpz being responsible for almost the entire AIDS pandemic (reviewed in ref. 2). Successful zoonosis usually requires species-specific adaptation of the virus to effectively interact with host cell factors that are essential for viral replication and transmission while concurrently avoiding or antagonizing innate and adaptive antiviral defenses. To this end, primate lentiviruses encode a number of accessory genes (*vpu*, *vif*, *vpr/vpx*, and *nef*) that play important roles in viral replication by encoding proteins that antagonize host restriction factors, contributing to evasion of antiviral defenses and manipulating the host cell environment (3, 4). Importantly, the contribution of these accessory genes to pathogenesis, species-specific adaptation, and successful zoonosis of lentiviruses has been increasingly realized (5).

The accessory protein Nef is particularly interesting in this regard. Nef is a small pleiotropic 27 to 35 kDa protein that promotes viral immune evasion and enhances virus infectivity by down-modulating multiple cellular proteins from the surface of infected cells (6). Nef is myristoylated and localizes to the plasma membrane, where it is abundantly expressed throughout the viral life cycle and interacts with multiple components of cell endocytic machinery, acting as an adaptor molecule to traffic protein targets toward the trans-Golgi network, endosomes, or lysosomes for redistribution and/or degradation (7). Specifically, Nef down-regulates CD4, MHC-I, CD28, and CXCR4 from the plasma membrane of infected cells to both enhance viral release and manipulate the antiviral host immune response (7, 8). Recently, Nef has also been shown to down-regulate the restriction factors SERINC3 and SERINC5 from infected cells, preventing their incorporation into nascent virions that would otherwise inhibit particle infectivity in an Env-dependent manner (9, 10). Notably, most SIVs and HIV-2 (the latter resulting from zoonotic transmissions of SIVsmm to humans) also use Nef to down-regulate expression of T cell receptor (TCR) component CD3ζ from the surface of infected cells (11). CD3 is responsible for T cell signal transduction following antigen-specific TCR binding to peptide–MHC displayed on an antigen-presenting cell, resulting in T cell activation and gain of effector function (reviewed in ref. 12). Thus, it has been proposed that CD3 down-regulation is an important immune evasion strategy of lentiviruses as it impairs immunological synapse formation (13, 14) and limits T cell activation (15) to dampen antiviral responses and apoptosis. Intriguingly, HIV-1 and its direct ancestor (SIVcpz) do not down-regulate CD3 and appear to have lost this Nef function twice during evolution, coincident with acquisition of *vpu* (11). Why HIV-1 and its precursors do not employ this potential immune evasion strategy remains incompletely understood. Although most SIV Nefs down-modulate CD3, Nef proteins that lost this function have been identified in SIVsmm-infected sooty mangabeys showing an unusual virological and immunological course of infection (15, 16). Specifically, these animals showed severe loss of CD4+ T cell counts but no clinical disease. Sequence and functional analyses identified mutations in Env that allowed efficient coreceptor usage of CXCR4 (in addition to CCR5, GPR15, and CXCR6, which are commonly used by SIVsmm) and subsequent changes in Nef that specifically disrupted its ability to down-regulate CD3 (16). Further in vitro analysis confirmed that the resulting retention of CD3 at the cell surface led to an increase in T cell activation and cell death following TCR cross-linking of infected CD4+ T cells (15, 16). However, whether these mutations in Nef directly affected the viral replication cycle or conferred any replicative advantage to the virus that may explain their selection in vivo, and by extension provide new insights into the loss of this Nef function by the HIV-1 lineage, remained to be fully defined.

In this study, we examined the effect of Nef-mediated regulation of CD3 on viral replication in primary human CD4+ T cells with the goal of defining viral parameters to explain the lineage-specific difference in Nef function. We report that viruses with Nefs that are unable to remove CD3 from the surface of infected primary T cells are more infectious and spread better between T cells than viruses containing Nefs that down-regulate CD3. Phenotypic and functional analysis showed that this increase in viral spread was associated with an increase in the abundance of Env trimers on the surface of infected cells and increased Env incorporation into virions but independent of SERINC5 antagonism. We thus demonstrate that loss of the CD3 down-modulation function of Nef is associated with a selective advantage, which helps to explain its manifestation in the primate lentiviruses that ultimately led to the emergence of HIV-1 and the AIDS pandemic.

## Results

### Retained CD3 Expression on Infected Cells Results in Increased Lentiviral Spread between Cells.

**Fig. 1.**
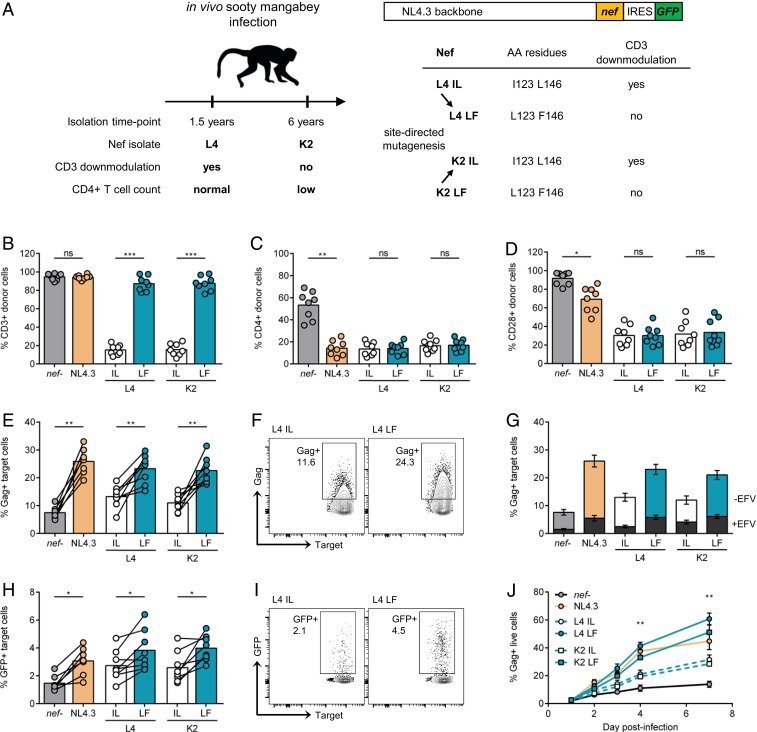
Retained CD3 expression on infected cells results in increased viral spread. (*A*) Nef mutant viruses used in this study. L4 IL (I123/L148) and K2 LF (L123/F148) *nef* alleles were isolated from an in vivo sooty mangabey infection and differ in their ability to down-modulate CD3. L4 LF and K2 IL *nef* alleles were created by site-directed mutagenesis. SIVsmm *nef* alleles or NL4.3 *nef* were inserted into replication competent NL4.3 backbone with an internal ribosome entry site (IRES)-driven GFP reporter gene. AA, amino acids. (*B*–*D*) Infected primary CD4+ T cells were analyzed by flow cytometry 48 h postinfection. Shown is the percentage of positive cells of total live Gag+GFP+ cells (donor cells) for (*B*) CD3, (*C*) CD4, and (*D*) CD28 surface markers. (*E*–*I*) Primary CD4+ T cells infected with indicated viruses were mixed with autologous prelabeled target cells and analyzed 24 h postmix by flow cytometry (*n* = 8). (*E*) Percentage of Gag+ target cells. (*F*) Representative flow cytometry plots from *E*. (*G*) Viral spread in the presence or absence of 5 μM efavirenz (±EFV). Superimposed gray bars show percentage of Gag+ targets in the presence of efavirenz (+EFV). (*H*) Percentage of GFP+ target cells. (*I*) Representative flow cytometry plots from (*H*). (*J*) CD4+ T cells were infected, and spreading infection was quantified by flow cytometry over time (*n* = 3). Bars show the mean, and lines join paired results from the same PBMC donor. Error bars show the mean ± SEM. Groups were compared using a two-tailed paired *t* test (not significant [ns], *P* > 0.05; *P < 0.05; **P < 0.01; ***P < 0.001).

To test whether loss of Nef-mediated CD3 down-regulation was associated with increased viral spread between T cells, we used a panel of previously described genetically engineered HIV-1 NL4.3 constructs coexpressing green fluorescent protein (GFP) and SIVsmm Nefs differing in this function from a bicistronic RNA (11, 16). As illustrated in Fig. 1*A*, these *nef* alleles were originally cloned from an SIVsmm-infected sooty mangabey that initially maintained normal CD4+ T cell levels (FBr 75wL4) but later exhibited profound CD4+ T cell loss (FBr 304wK2) (15, 16), hereafter abbreviated as L4 and K2, respectively. Nef sequence analysis identified two specific amino acid changes (I123L and L146F) that specifically disrupted the CD3 down-modulation activity (16). Corresponding gain or loss of function mutants of L4 (L123/F146) and K2 (I123/L146) were generated by site-directed mutagenesis (16). For simplicity, we hereafter collectively refer to viruses that retained CD3 down-regulating activity of Nef as I123/L146 (abbreviated to IL) and those that lost CD3 down-regulating activity as L123/F146 (abbreviated to LF) (Fig. 1*A*). It has previously proven impossible to engineer HIV-1 Nef capable of down-regulating CD3 without disrupting other functions of Nef, including CD4, CD28, CXCR4, and MHCI down-modulation as well as CD74 up-regulation (17). Inserting these naturally occurring SIVsmm *nef* alleles into the HIV-1 NL4.3 molecular clone allowed us to directly test the impact of this change in Nef function on HIV-1 spread in a background where all other genes were identical.

To validate the panel of viruses, primary CD4+ T cells were infected with Nef-expressing or the *nef-*defective viruses, and surface expression of CD3, CD4, and CD28 on T cells was measured by flow cytometry (Fig. 1 *B*–*D*). Infected T cells were identified as double positive for intracellular Gag staining and GFP expression, coupled with live–dead staining (*SI Appendix*, Fig. S1). As expected from published data (16, 18), all HIV-1 Nefs (NL4.3 and two primary HIV-1 Nefs, NA7 and SF2), as well as the *nef-*defective virus, did not down-modulate CD3 (Fig. 1*B* and *SI Appendix*, Fig. S2). Of the viruses containing SIVsmm *nef* alleles, L4 and the K2 IL Nefs down-modulated CD3, while the parental K2 and the L4 LF Nefs lacked this function (Fig. 1*B*). By contrast, all parental and mutant *nef* viruses had similar effects on CD4 and CD28 (Fig. 1 *C* and *D*). Partial CD4 down-modulation observed in the absence of Nef is attributed to the presence of HIV-1 Vpu that mediates CD4 degradation (19).

To determine the effect of CD3 down-modulation on viral replication and spread, we performed a well-established assay in which HIV-1–infected primary CD4+ T cells (donor cells) are cocultured with prelabeled, uninfected autologous primary CD4+ T cells (targets) (refs. 20–23 and *SI Appendix*, Fig. S1). This assay primarily measures cell-to-cell spread (which is the dominant mode of HIV-1 dissemination) with minimal contribution from cell-free virus infection (20–24) or cell-to-cell fusion (25). Primary CD4+ T cells were activated and infected with equivalent doses of infectious virus to achieve similar numbers of infected cells (*SI Appendix*, Fig. S1). The percentage of infected donor cells was quantified by flow cytometry at 48 h postinfection, and these cells were then mixed at a 1:4 ratio with autologous dye-labeled primary CD4+ target T cells (*SI Appendix*, Fig. S1). After 24 h of coculture (which restricts replication to a single round), flow cytometry was performed to identify newly infected Gag+ and GFP+ cells target T cells. As expected, all viruses encoding Nef (NL4.3 wild-type [WT], NA7, SF2, IL, and LF variants) spread significantly more efficiently than *nef-*defective virus (Fig. 1*E* and *SI Appendix*, Fig. S2). Notably, we found that L4 and K2 LF Nef viruses, which do not remove CD3 from the surface infected cells, spread significantly better by cell-to-cell spread when compared to L4 and K2 IL Nef viruses that down-modulate CD3 (Fig. 1 *E* and *F*). Treating donor and target cell cocultures with the reverse transcriptase inhibitor efavirenz resulted in a fivefold reduction in the number of Gag+ target cells, confirming that the signal measured in target cells comes from de novo Gag synthesis that is indicative of productive infection (Fig. 1*G*). Consistent with these observations, retained CD3 expression also correlated with better cell-to-cell spread when infection was quantified by the expression of the long-terminal repeat (LTR)-driven GFP in target T cells (Fig. 1 *H* and *I*). Furthermore, measuring spreading infection between T cells over a longer time course confirmed that viruses that did not down-regulate CD3 (L4 IL and K2 LF) showed accelerated replication kinetics (Fig. 1*J*), consistent with the expression of CD3 on infected donor T cells allowing more efficient viral transmission to target T cells.

### Retained CD3 Expression Correlates with Increased Cell Activation and Death.

**Fig. 2.**
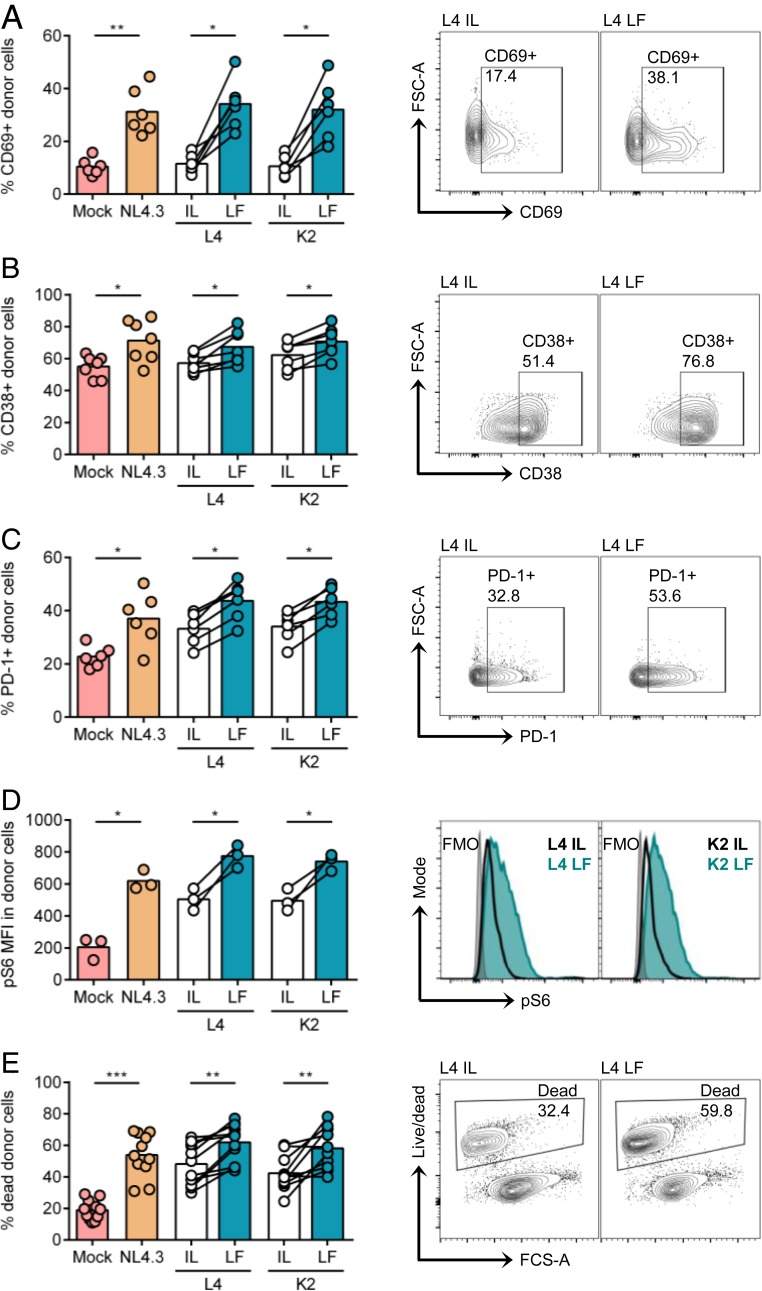
Retained CD3 expression results in increased cell activation and cell death during cell-to-cell spread. Primary CD4+ T cells infected with indicated viruses were mixed with autologous prelabeled target cells and analyzed by flow cytometry 24 h postmix as described in Fig. 1. (*A*–*D*) Infected (Gag+GFP+) donor cell populations (donor cells) were analyzed for cell surface expression of activation markers. Mock is uninfected cells, and total donor live population was analyzed. (*A*) Percentage of CD69+ donor cells. (*B*) Percentage of CD38+ donor cells. (*C*) Percentage of PD-1+ donor cells. (*D*) MFI of phosphorylated S6 (pS6) in donor cells. (*E*) Percentage of dead cells in total donor cell population. Shown are representative flow cytometry plots or histograms of data in *A*–*E*. Bars show the mean, and lines join paired results from the same PBMC donor. Groups were compared using a two-tailed paired *t* test (*P < 0.05; **P < 0.01; ***P < 0.001).

Cell-to-cell spread confers an advantage to HIV-1 by increasing the kinetics of viral replication and spread (20–24). Notably, cell-to-cell spread has also been associated with activation of T cell signaling in the HIV-1 infected cell that we showed is mediated in part by the TCR/CD3 complex (26). Thus, we explored whether cell-to-cell spread activated signaling and accelerated cell death in infected donor cells in a CD3- and Nef-dependent manner. Flow cytometry analysis of infected donor CD4+ T cells following coculture with uninfected targets (to allow for cell-to-cell contact) showed that retained CD3 expression (NL4.3 WT, L4 LF, and K2 LF) correlated with significantly increased expression of the activation markers CD69, CD38, and PD-1 and phosphorylation of the downstream ribosomal protein S6 (Fig. 2 *A*–*D*). Moreover, cell death was also increased compared to cells infected with viruses that down-modulated CD3 (L4 IF and K2 IF) or to uninfected cells (Fig. 2*E*). Activation of T cell signaling during cell-to-cell spread also correlated with increased viral gene expression, as evidenced by an increase in LTR-driven GFP MFI and Gag MFI in cells infected with viruses that retained CD3 expression (*SI Appendix*, Fig. S3). We conclude that in addition to driving more efficient viral dissemination during cell-to-cell spread, retention of CD3 on infected primary T cells may also promote contact-induced T cell activation and cell death.

### Retained CD3 Expression Is Associated with Increased Env Incorporation into Virions.

**Fig. 3.**
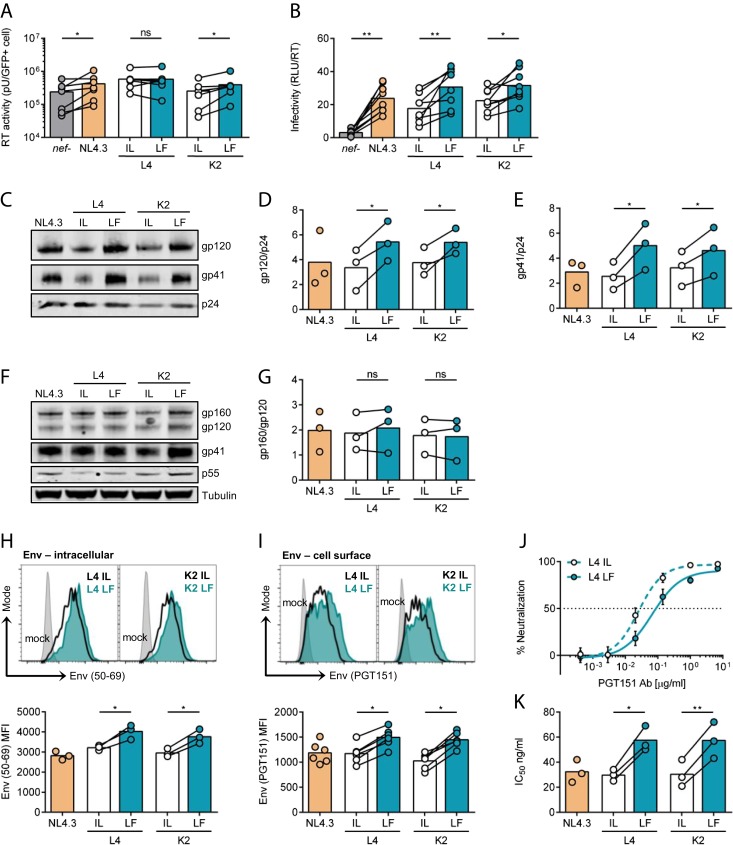
CD3 expression on infected cells increases viral infectivity by increasing Env incorporation into virions. (*A* and *B*) Primary CD4+ T cells were infected with indicated viruses, and culture supernatants were collected at 48 h postinfection (*n* = 8). (*A*) Virus release measured by supernatant RT activity and normalized to the number of infected GFP+ cells. (*B*) Virion infectivity (RLUs) normalized to supernatant RT activity from (*A*). (*C*–*E*) Western blotting of virus purified from infected primary CD4+ T cell supernatants (*n* = 3). (*C*) Representative immunoblot shows detection of Env gp120, Env gp41, and Gag p24. (*D*) Quantification of gp120 incorporation normalized to p24. (*E*) Quantification of gp41 incorporation normalized to p24. (*F* and *G*) Western blotting of infected primary CD4+ T cell lysates (*n* = 3). (*F*) Representative immunoblot shows detection of Env gp160, Env gp120, Env gp41, Gag p55, and tubulin. (*G*) Quantification of Env cleavage, shown as the ratio of gp160/gp120. (*H* and *I*) Flow cytometry analysis of CD4+ T cells infected with indicated viruses measuring expression of (*H*) intracellular Env (*n* = 3) and (*I*) cell surface Env (*n* = 6). *Upper* plots show representative histograms, and *Lower* plots show combined data from multiple independent experiments. (*J* and *K*) Neutralization of virus (produced from primary CD4+ T cells) by PGT151 Ab (*n* = 3). (*J*) Representative examples of neutralization curves. (*K*) IC_50_ values calculated by nonlinear regression analysis of the neutralization curves. Bars show the mean, and lines join paired results from the same PBMC donor. Error bars show mean ± SD. Groups were compared using a two-tailed paired *t* test (not significant [ns], *P* > 0.05; *P < 0.05; **P < 0.01).

To determine how Nef-mediated regulation of CD3 surface expression modulates viral spread, primary CD4+ T cells were infected with different Nef-expressing viruses, supernatants were harvested, and viral budding and infectivity were measured. Quantification of viral reverse transcriptase (RT) activity in cell supernatants (a marker of viral content) revealed no marked difference associated with CD3 down-regulation, indicating that all *nef* alleles allow budding from infected primary T cells to similar levels (Fig. 3*A*). By contrast, titrating viral supernatants on HeLa TZM-bl reporter cell lines and normalizing the relative light units (RLU) to viral output (RT activity) showed that virions produced from infected cells that retained CD3 (L4 and K2 LF) had significantly increased particle infectivity (Fig. 3*B*). Increased cell-to-cell spread was not mediated by enhanced VS formation between primary CD4+ T cells (*SI Appendix*, Fig. S4), further implicating increased virion infectivity.

A major determinant of lentiviral infectivity is the envelope glycoprotein (Env) that is incorporated into virions and binds to the entry receptors CD4 and the coreceptors CXCR4 or CCR5 (reviewed in ref. 27). Env is synthesized as a gp160 precursor that undergoes proteolytic cleavage by cellular furin to produce a functional protein consisting of the gp120 receptor binding subunit and the gp41 subunit that mediates viral fusion (reviewed in ref. 27). To gain insight into why a virus produced in the presence of CD3 showed increased infectivity, we analyzed Env processing, expression, and virion incorporation. Notably, Western blotting of purified virions revealed that viruses retaining CD3 on infected cells (L4 LF and K2 LF) incorporated significantly more Env gp120 and gp41 into nascent particles compared to viruses that down-modulated CD3 (L4 IL and K2 IL) (Fig. 3 *C*–*E*). This was not simply due to increased furin-mediated processing of Env gp160 since Western blotting of cell lysates showed no difference in the ratio of unprocessed (gp160) to processed (gp120) Env (Fig. 3 *F* and *G* and *SI Appendix*, Fig. S5 *A*–*D*). More quantitative flow cytometry analysis revealed that cells infected with LF Nef-expressing viruses showed increased levels of intracellular and cell surface Env on infected cells as depicted by an increase in Env MFI (Fig. 3 *H* and *I* and *SI Appendix*, Fig. S5*E*). To determine if differences in Env retention or internalization from the plasma membrane were also implicated in altered Env surface expression and incorporation into virions, a flow cytometry Env internalization assay (28) and an Env recycling assay were performed (*SI Appendix*, Fig. S6). These data show that the kinetics of Env internalization was faster in cells infected with viruses that retain CD3 (L4 and K2 LF). Similarly, retention of CD3 (L4 and K2 LF) resulted in faster kinetics of Env recycling back to the cell surface. These data suggest that while CD3 does not retain Env at the cell surface to enhance incorporation into virions, the presence of CD3 appears to influence cellular pathways that regulate Env trafficking to and from the cell surface that may in turn impact subsequent Env incorporation into nascent virions.

Because Env in virions can exist in multiple states, including cleaved functional trimers that are competent for receptor binding and fusion, and nonfunctional forms (monomers, dimers, noncleaved gp160, and nonfunctional gp41 stumps) (29), we sought to confirm that the increase in viral infectivity and Env incorporation (Fig. 3 *B*–*E*) correlated with more functional Env in virions. To do this, we performed neutralization assays using two different HIV-1 broadly neutralizing antibodies (Abs) that specifically recognize functional Env timers: PGT151 (cleaved Env trimers) (30) and PG9 (the Env trimer apex) (31). Virions produced in the presence of L4 and K2 LF Nefs (CD3 retention and increased infectivity) required more antibody to neutralize infection by 50% and thus displayed significantly higher 50% inhibitory concentration IC_50_ values when compared to virions encoding the L4 and K3 IL Nef proteins (Fig. 3 *J* and *K* and *SI Appendix*, Fig. S5 *F* and *G*). This increase in the IC_50_ is consistent with more Env trimers being incorporated into the virions that in turn require more antibody to be neutralized.

### Increased Infectivity Is Independent of Nef Antagonism of SERINCs.

Nef has been reported to increase lentiviral infectivity by antagonizing the host restriction factor SERINC5 (9, 10). Specifically, Nef removes SERINC5 and SERINC3 from sites of viral budding at the plasma membrane, thereby preventing their incorporation into nascent particles that would otherwise inhibit viral infectivity (10). To exclude the possibility that increased infectivity and cell-to-cell spread was attributable to differences in the ability of these Nef proteins to antagonize SERINC5, HEK293T cells were cotransfected with viral plasmids alongside increasing doses of plasmid-encoding SERINC5 that was dual tagged with an intracellular HA tag and an extracellular FLAG tag (*SI Appendix*, Fig. S7). Dose-dependent increases in SERINC5 expression were confirmed by flow cytometry staining for HA-SERINC5 (*SI Appendix*, Fig. S7*A*). Consistent with SERINC5 inhibiting HIV-1 infectivity in a Nef-dependent manner, we observed up to a 25-fold reduction in infectivity of *nef*-defective virus produced in the presence of increasing doses of SERINC5 that was rescued by expression of functional Nef (*SI Appendix*, Fig. S5*B*). High doses of SERINC5 also inhibited NL4.3 Nef-expressing viruses due to SERINC5 overexpression overwhelming endogenous Nef activity. The inability of *nef*-defective virus to antagonize SERINC5 (*SI Appendix*, Fig. S7*B*) (9, 10), alongside other reported effects of Nef on Gag biosynthesis (32), likely explains why *nef*-defective virus showed reduced cell-to-cell spread (Fig. 1 *E* and *H*) and reduced virion infectivity (Fig. 3*B*) when compared to NL4.3 WT, IL, and LF Nef variants. Importantly, IL and LF viruses showed no difference in their ability to antagonize SERINC5 to enhance viral infectivity (*SI Appendix*, Fig. S7*B*) and were equally efficient in down-regulating SERINC5 from the cell surface as measured by staining for extracellular FLAG-tagged SERINC5 (*SI Appendix*, Fig. S7*C*). Similar results were obtained in the presence of overexpressed SERINC3 (*SI Appendix*, Fig. S7 *D*–*F*). Taken together, these data show that residues in Nef that determine CD3 down-modulation by binding to CD3ζ do not affect anti-SERINC activity and that differences in IL and LF viral infectivity cannot be explained by differences in the Nef variants’ anti-SERINC activity or sensitivity to SERINC-mediated inhibition.

### RNAi Depletion of CD3 in Primary T Cells Reduces Viral Infectivity.

**Fig. 4.**
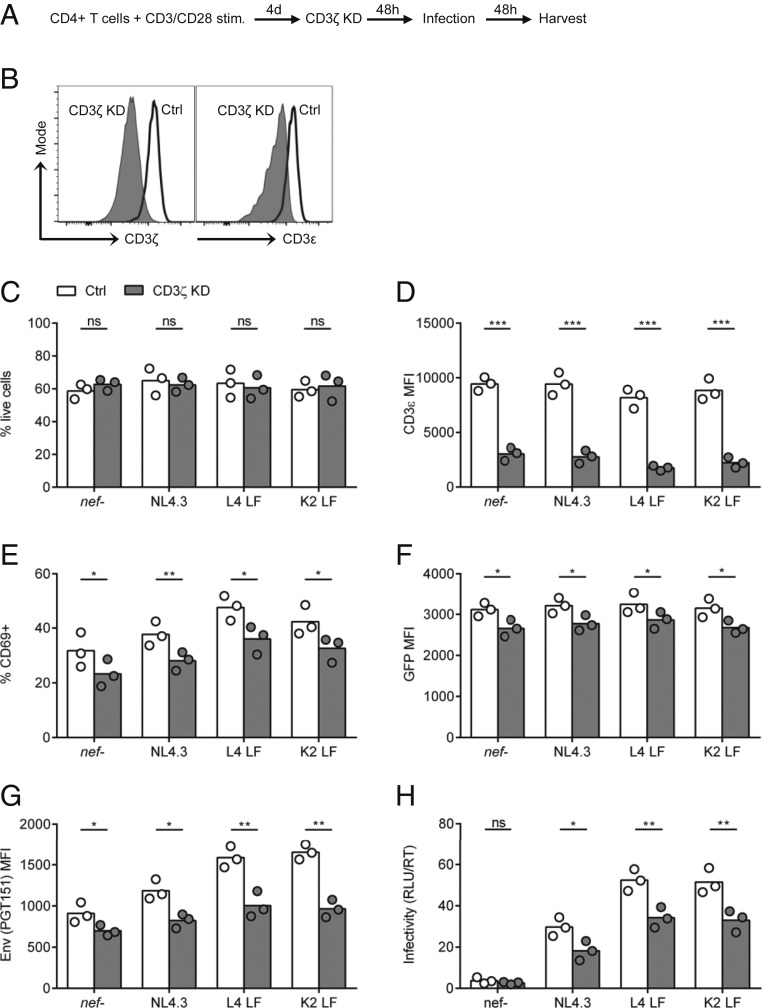
CD3 knockdown reduces surface Env expression and virion infectivity. (*A*) Activated primary CD4+ T cells were electroporated with siRNA targeting CD3ζ (CD247) or a nontargeting control for 48 h and infected with indicated viruses (*n* = 3). (*B*) Histograms show flow cytometry analysis for intracellular expression of CD3ζ (CD247) and surface expression of CD3ε at 48 h postknockdown. (*C*) RNAi knockdown does not affect cell viability at 48 h postinfection. (*D*–*G*) Flow cytometry analysis for expression of (*D*) CD3ε, (*E*) CD69, (*F*) GFP, and (*G*) Env (PGT151) on infected GFP+ cells at 48 h postinfection. (*H*) Virion infectivity at 48 h postinfection was quantified as described in Fig. 3. Bars show the mean, and symbols show individual PBMC donors. Groups were compared using a two-tailed paired *t* test (not significant [ns], *P* > 0.05; *P < 0.05; **P < 0.01; ***P < 0.001).

To examine whether the increase in functional Env trimers and enhanced viral infectivity was directly attributable to Nef regulation of CD3 expression (and not due to some other as yet undefined function of residues 123 and 146 in Nef), we used small interfering RNA (siRNA) to deplete CD3ζ (CD247) in primary CD4+ T cells (Fig. 4*A* and *SI Appendix*, Fig. S8). Flow cytometry analysis confirmed the reduction in CD3ζ expression as well as a corresponding reduction in CD3ε (Fig. 4*B*). CD3 depletion did not affect cell viability (Fig. 4*C*) and was maintained in the infected cell population 48 h postinfection (Fig. 4*D*). Primary CD4+ T cells depleted of CD3 and infected with viruses that are unable to down-modulate CD3 (NL4.3, L4 and K2 LF) showed a reduction in cellular activation (measured by CD69 expression) and viral gene expression (measured by GFP MFI) (Fig. 4 *E* and *F* and *SI Appendix*, Fig. S8*D*). Moreover, RNA interference knockdown (RNAi KD) of CD3 also correlated with a significant reduction in the amount of Env trimer (PGT151) staining on the plasma membrane of infected cells and reduced virion infectivity compared to cells treated with control siRNA (Fig. 4 *G* and *H*). CD3 KD in cells infected with viruses that can down-modulate CD3 (L4 and K2 IL) showed an additive effect that can be explained by a further reduction in CD3 MFI in these cells (*SI Appendix*, Fig. S8*B*). Thus, depleting CD3 phenocopied what was observed following Nef-mediated CD3 down-regulation. Taken together, these data show that increased expression of functional Env on the surface of infected cells leads to increased incorporation into virions, in turn mediating enhanced infectivity that leads to better viral spread to target cells in a Nef- and CD3-dependent manner.

### CD3-Mediated Enhancement of Virion Infectivity and T Cell Activation.

**Fig. 5.**
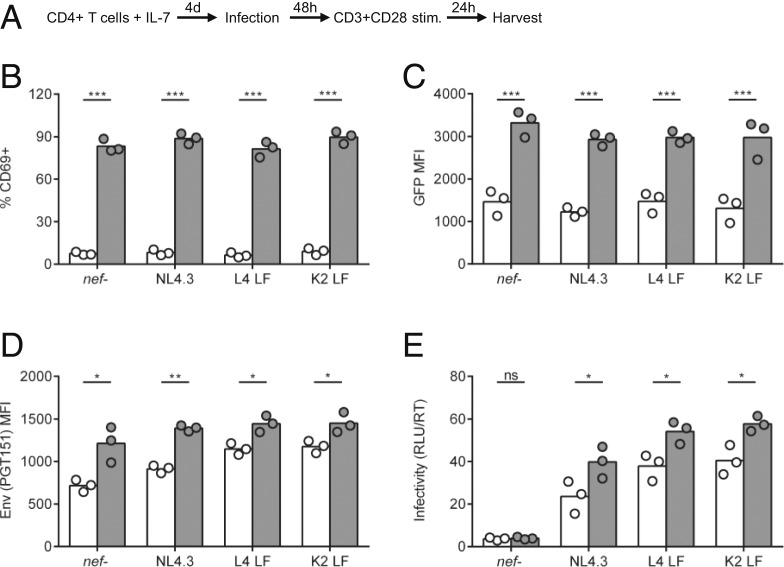
Env expression and virion infectivity is increased by T cell activation. (*A*) Resting primary CD4+ T cells were treated with IL-7 for 4 d and then infected with indicated viruses; 48 h postinfection, cells were stimulated with anti-CD3/CD28 antibodies for 24 h or left untreated (mock) (*n* = 3). (*B*–*D*) At 24 h poststimulation cells were analyzed by flow cytometry for (*B*) expression of CD69 in total live population, (*C*) expression of GFP in infected GFP+ population, and (*D*) expression of Env (PGT151) in infected GFP+ population. (*E*) Virion infectivity at 48 h postinfection was quantified as described in Fig. 3. Bars show the mean, and symbols show individual PBMC donors. Groups were compared using a two-tailed paired *t* test (not significant [ns], *P* > 0.05; *P < 0.05; **P < 0.01; ***P < 0.001).

In vivo, the TCR/CD3 complex drives T cell activation following stimulation by an antigen-presenting cell. As such, retaining CD3 expression could make HIV-1 infected primary T cells more responsive to stimulation, thereby boosting viral gene expression and replication. To explore this, resting primary CD4+ T cells were treated with IL-7 to make them permissive to HIV-1 infection without T cell activation (33), infected with viruses that cannot down-regulate CD3 (NL4.3, L4 LF, and K2 LF), and subsequently stimulated with anti-CD3/CD28 antibodies to induce TCR triggering and activation (Fig. 5*A* and *SI Appendix*, Fig. S9). T cell activation of infected resting cells was confirmed by up-regulation of the activation marker CD69 on 90% of stimulated cells (Fig. 5*B*) and increased proviral transcription measured by LTR-driven GFP-expression (Fig. 5*C*). Inducing robust T cell activation by CD3 cross-linking of HIV-1 infected resting primary T cells significantly increased the expression of Env trimers on the surface of infected cells and virion infectivity (Fig. 5 *D* and *E*). The fact that this effect was seen across all Nef viruses, including CD3–down-modulating IL Nef viruses (*SI Appendix*, Fig. S9), can be explained by a proportion of the IL Nef-infected cells still expressing CD3 at the time of stimulation, meaning they had yet to down-regulate CD3 and were thus able to respond to stimulation.

## Discussion

Here, we have interrogated the consequence of differences in Nef-mediated CD3 down-regulation for HIV-1 replication and have shown that the inability of Nef to remove CD3 from infected CD4+ T cells increases HIV-1 spread between T cells. With few exceptions [e.g., Nef- vs. Vpu-mediated antagonism of tetherin (34–36)], Nef functions are conserved across different lentiviral lineages, highlighting the importance of both viral manipulation of the host cell environment and the dependence on accessory proteins for lentiviral replication. However, a key question has remained incompletely understood in HIV-1 biology: If virus-induced down-regulation of CD3 from the surface of infected cells confers an important immune evasion strategy, is there any selective advantage for HIV-1 not to do this? We now show that HIV-1 engineered to down-regulate CD3 does not replicate and spread as efficiently between T cells as HIV-1 that retains CD3, the latter representing the normal phenotype of HIV-1 Nef, its simian ancestor SIVcpz/gor, and closely related SIVgsn/mus/mon (11). Detailed examination of virion assembly, budding, infectivity, and replication identified increased Env expression at the cell surface and incorporation into virions in the presence of CD3 as the determinant for better viral spread. Env is a limiting factor in viral infectivity due to the presence of just a few functional Env trimers on virions (37). This likely explains why increasing the number of Env trimers on the surface of infected cells and incorporation into viral particles correlated with greater infectivity and better viral cell-to-cell spread. Furthermore, CD3-mediated stimulation of infected T cells increased T cell activation, Env surface expression, virion incorporation, and infectivity (Fig. 5), consistent with CD3 engagement mediating T cell signaling, T cell activation, and intracellular remodeling. Interestingly, analysis of Env trafficking revealed a small but significant difference in the kinetics of Env internalization and recycling back to the plasma membrane between viruses that did and did not down-regulate CD3 that may in turn contribute to Env incorporation. For example, it was recently reported that efficient incorporation of Env into nascent viral particles requires Env endocytosis and the redelivery of Env to specific virus assembly domains at the plasma membrane (38). Furthermore, perturbing postendocytic sorting has been shown to influence the amount of Env incorporated into viral particles (38–40). Integrating these observations suggests that retaining CD3 on the surface of HIV-1–infected primary CD4+ T cells may influence the dynamics of Env trafficking to and from the cell surface and thus virion incorporation. Gaining a better understanding of the host factors that regulate Env trafficking and incorporation into virions will help shed light on the role of CD3 and T cell signaling in this process. Cell-to-cell contact between HIV-1–infected and -uninfected T cells during viral spread can activate components of T cell signaling, including the TCR/CD3 complex on infected donor cells, through stimulatory interactions between cells (26). Thus, increased Env-dependent viral infectivity and spread may be triggered either by classical T cell activation following antibody-mediated CD3 cross-linking or by activation of signaling during coculture of infected and uninfected CD4+ T cells. Limiting the levels of functional Env trimers displayed on infected T cells and incorporated into virions until a susceptible target T cell was available to stimulate the donor cell via cell-to-cell contact would be an elegant way to limit excessive exposure of Env to the humoral immune system, including neutralizing antibodies. This is an intriguing scenario that warrants further study.

The loss of CD3 down-regulation by Nef has occurred twice in the lentiviral lineages that gave rise to pandemic HIV-1, coincident with the acquisition of *vpu* (reviewed in ref. 5), with the presence of *vpu* being specific to HIV-1 and some closely related simian lentiviruses (11). This has led to the notion that loss of Nef-mediated CD3 down-regulation was facilitated by the acquisition of a *vpu* gene in a subset of primate lentiviruses, including HIV-1 (41, 42). Vpu suppresses T cell activation by interfering with NF-κB to limit interferon-stimulated gene induction and innate immune responses (41, 43–45), in addition to antagonizing the restriction factor tetherin (46) and mediating CD4 degradation. By contrast, Nef stimulates IKKβ-mediated NF-κB activation to boost viral LTR activity and increase proviral transcription (43). This creates an intriguing challenge for the virus of temporally balancing two opposing activities of Nef and Vpu to drive efficient viral transcription and replication while concomitantly suppressing antiviral responses and is comprehensively reviewed in ref. 41. The requirement to toggle T cell activation during different stages of the viral replication cycle likely explains why acquisition of *vpu* correlated with loss of Nef-mediated CD3 down-regulation (41); however, it does not explain why keeping CD3 should be better. Here, we provide an explanation for why loss of this Nef function is advantageous by showing that CD3 enhances Env trimer expression at the cell surface and incorporation into virions, leading to increased viral infectivity and spread. The replicative advantage to the virus for retaining CD3 goes some way to explaining the selective pressure for loss of this Nef function by the *vpu*-containing HIV-1 lineage. Specifically, our data support the notion that HIV-1 evolved to balance two opposing activities of lentiviral accessory proteins in CD4+ T cells by targeting NF-κB via Vpu, instead of CD3 via Nef, to suppress immune activation while allowing for efficient viral spread between cells.

What might be the effects of lineage-specific differences in lentiviral Nef function on viral pathogenesis? While it is difficult to extrapolate to in vivo, it is tempting to speculate of a possible link between retained CD3 expression and increased viral pathogenesis for some lentiviral lineages. For example, inefficient Nef-mediated down-modulation of CD3 correlated with low numbers of CD4+ T cells in SIVsmm-infected sooty mangabeys (15, 16) and has been observed in viremic HIV-2 infected individuals (47). Notably, the SIVsmm Nef protein K2 that lost the ability to down-regulate CD3 was isolated from a sooty mangabey that showed a profound loss of CD4+ T cells (15, 16). Whether the loss of CD4+ T cells, associated with accelerated CD4+ T cell activation and cell death, was influenced by changes in Nef-mediated CD3 down-regulation was unclear. Our results show that retained CD3 expression increases both viral replication and activation-induced cell death. Based on these data, it is plausible that CD3-dependent activation and cell death upon cell-to-cell contact in lymphoid tissue (where T cells are densely packed and interact) may have contributed to the accelerated loss of CD4+ T cells in these animals. Notably, selective disruption of Nef-mediated CD3 down-modulation was recently shown to increase immune activation and antiviral gene expression during in vivo infection with SIVmac239 in rhesus macaques, leading to enhanced inflammatory responses (48). Conversely, activation of antiviral immune responses and inflammation may lead to improved control of viral replication. Sooty mangabeys do not progress to AIDS, despite high viral loads, and this has been attributed to species-specific host factors rather than viral virulence (49). By contrast, infections of humans with HIV-1 and chimpanzees with the simian ancestor SIVcpz are associated with loss of CD4+ T cells and disease (50–52). Both HIV-1 and SIVcpz are zoonotic transmissions into nonnatural hosts, the latter likely originating from a recombination between precursors of SIVgsn/mon/mus and SIVrcm (reviewed in ref. 5). In the context of zoonoses and viral adaptation to the new human host, increased HIV-1 replication fitness would be advantageous and selectively favored, perhaps even at the expense of additional means of immune evasion and the consequence of increased pathogenicity.

## Materials and Methods

### Cells and Viral Constructs.

Peripheral blood mononuclear cells (PBMCs) were isolated from buffy coats from anonymous, healthy donors (provided by UK National Health Service Blood and Transplant Service) using Ficoll Paque Plus (Sigma) according to manufacturer’s instructions. CD4+ T cells were isolated by negative selection using MojoSort Human CD4+ T Cell Isolation kit (Biolegend). Primary CD4+ T cells were maintained in RPMI medium 1640 (Thermo Fisher Scientific) supplemented with 10% fetal calf serum (FCS) (Labtech), 100 U/mL penicillin–streptomycin (Thermo Fisher Scientific), and 10 IU/mL IL-2 (Centre for AIDS Reagents [CFAR], National Institute of Biological Standards and Controls [NIBSC]). HEK293T and HeLa TZM-bl cell lines were grown in Dulbecco’s modified Eagle’s medium (Thermo Fisher Scientific) supplemented with 10% FCS and 100 U/mL penicillin–streptomycin. Proviral constructs carrying functional *nef* alleles and *nef*-defective virus were previously described (16). Viral stocks were generated by transfecting HEK293T cells using Fugene 6 (Promega). After 48 h, virus was harvested, purified by sucrose gradient ultracentrifugation, and titrated by infectivity assay on HeLa TZM-bl reporter cells using Bright-Glo (Promega).

### HIV-1 Infections and Cell-to-Cell Spread Assays.

Primary CD4+ T cells were activated for 4 to 5 d on T25 flasks coated with 5 μg anti-CD3 antibody (clone OKT3, Biolegend) in the presence of 2 μg/mL soluble anti-CD28 antibody (clone CD28.2, Biolegend). Cells were infected with HIV-1 by spinoculation (1,200 × *g* for 2 h at RT) at a multiplicity of infection of 0.1. For cell-to-cell spread assays HIV-1–infected T cells (donors) were analyzed for GFP expression by flow cytometry 48 h postinfection (p.i.), and the number of GFP+ cells was measured using Precision Count Beads (Biolegend). Uninfected T cells (targets) were labeled with 2.5 μM Cell Proliferation Dye eFluor 450 (Thermo Fisher Scientific) according to manufacturer’s instructions. HIV-1–infected GFP+ donor cells were normalized to achieve equivalent numbers of infected cells (10%) to account for variability between different PBMC donors in initial infection rates and then mixed with dye-labeled targets at a 1:4 ratio, incubated at 37 °C for 24 h, and analyzed by flow cytometry. For longer-spreading infection assays, donor cells were analyzed for GFP expression by flow cytometry at 24 h p.i., infection levels were adjusted to 2% with uninfected CD4+ T cells, and cells were incubated at 37 °C and analyzed by flow cytometry at various time points.

### Virus Release and Infectivity Assays.

HIV-1–infected CD4+ T cells were incubated for 48 h, and virus culture supernatants were harvested. Total virus released into the supernatant was measured by qPCR to quantify the supernatant RT activity using SYBR-green–based product enhanced reverse transcription assay (53), and RT activity was normalized to the number of GFP+ (HIV-1–infected) cells. Virion infectivity was determined by luciferase assay using HeLa TZM-bl reporter cells. To determine particle infectivity, RLU infectivity values were normalized to RT activity.

### Flow Cytometry.

T cells were washed and incubated with antibodies at 4 °C for surface staining (see *SI Appendix* for antibodies) and cell viability dye Zombie UV (Biolegend). Primary antibodies were detected with anti-human IgG secondary antibody, fixed, and analyzed. To stain for intracellular antigens, cells were fixed with 4% paraformaldehyde and permeabilized with CytoPerm buffer (Biolegend). Cells were analyzed on a BD LSR Fortessa X-20 cytometer. Compensation was performed using CompBeads (BD) and calculated by FACSDiva software. Data were analyzed using FlowJo software.

### Western Blotting.

Twenty micrograms of cell lysate and an equal volume of purified virus were separated by sodium dodecyl sulfate–polyacrylamide gel electrophoresis and analyzed by Western blotting using the following primary antibodies: anti–HIV-1 gp120, anti–HIV-1 p41, anti–HIV-1 Gag, and anti-tubulin (see *SI Appendix* for antibodies). Primary antibodies were detected with appropriate fluorescent secondary antibodies and imaged with an Odyssey Infrared Imager (Licor). Immunoblots were analyzed with Image Studio Lite software.

### Immunofluorescence Microscopy.

Uninfected CD4+ T cells (target cells) were labeled with 10 μM CellTracker Blue CMAC dye (Thermo Fisher Scientific) according to the manufacturer’s instructions. Infected CD4+ T cells (donor cells) were mixed with target cells in a 1:1 ratio and incubated with anti-Env Ab (nonblocking) for 1 h at 37 °C on poly-l-lysine–coated coverslips. Cells were fixed with 4% paraformaldehyde and permeabilized with 0.1% Triton X-100. Samples were stained with anti-Gag Ab. Primary antibodies were detected with appropriate fluorescent secondary antibodies. Coverslips were mounted with ProLong Gold Antifade mounting solution (Thermo Fisher Scientific). Images were acquired on a DeltaVision Elite image restoration microscope (Applied Precision) with softWoRx 5.0 software. Image processing was performed using Huygens Professional 4.0 and Adobe Photoshop 7 software. Virological synapse was defined as a donor–target cell conjugate with Gag and Env polarized toward the cell-to-cell contact as defined previously (20).

### Env Internalization Assay.

Env internalization assay was performed as described previously (28). Briefly, infected CD4+ T cells were stained with Env PGT151 Ab (5 μg/mL) and cell viability dye (Zombie UV) for 30 min on ice and then extensively washed in cold buffer. To start Env internalization, cells were incubated in complete culture media at 37 °C for 0 to 120 min and then fixed in 2% paraformaldehyde. For T_0min_ (time = 0 min) control cells were fixed after Env staining, without incubation at 37 °C. The remaining Ab–Env complexes on the cell surface were detected with anti-human IgG antibody, and cells were analyzed by flow cytometry.

### Env Recycling Assay.

Infected CD4+T cells were stained with Env PGT151 Ab (5 μg/mL) for 60 min at 37 °C in complete culture media to allow for Env internalization. Cells were extensively washed in cold buffer, and surface Env was stained with anti–human-PE secondary antibody (and cell viability dye) for 30 min on ice to measure baseline (T_0min_) surface Env expression. Cells were then extensively washed in cold buffer and incubated with anti-human-Cy5 secondary antibody for 0 to 120 min at 37 °C in complete culture media to label Env that was being recycled back to the cell surface. Cells were fixed in 2% paraformaldehyde and analyzed by flow cytometry.

### SERINC Antagonism Assay.

SERINC5 and SERINC3 expression vectors (pcDNA based) were gifted by Massimo Pizzato, University of Trento, Trento, Italy (54). SERINC proteins were HA tagged at the N terminus (intracellular tag) and FLAG tagged in the fourth extracellular loop. 293T cells were seeded in 24-well plates (2 × 10^5^/well) and transfected with 120 ng proviral DNA, 0 to 20 ng SERINC DNA, and empty pcDNA vector to equalize DNA content in 0.5 mL culture medium. Transfected cells and virus-containing supernatants were collected at 48 h posttransfection. Virion infectivity was measured as described above. Cells were analyzed for SERINC expression by flow cytometry.

### Neutralization Assays.

Antibody neutralization assay was performed as described previously (55). Briefly, virus supernatants were incubated with serial dilutions of PG9 (31) or PGT151 (30) antibodies (gift from Laura McCoy, University College London, London, UK) and incubated at 37 °C for 1 h. The mixture was added to HeLa TZM-bl reporter cells, and luciferase activity was measured 48 h later using Bright-Glo substrate. Antibody neutralization was calculated as percent decrease in luciferase activity compared to the corresponding virus-only control. IC_50_ values were calculated by nonlinear regression analysis (sigmoid curve interpolation) using Prism software (GraphPad).

### RNAi Knockdown.

CD247 (CD3ζ) was knocked down using ON-TARGET plus Human CD247 siRNA - SMARTpool (Dharmacon). Nontargeting siRNA (Dharmacon) was used as a control. CD4+ T cells were activated for 4 d as described above, and 2 × 10^6^ cells were electroporated with 250 pmol siRNA using a Neon Transfection System (Thermo Fisher Scientific; three pulses, 10 ms, 1,600 V). After 48 h, cells were analyzed by flow cytometry and infected as described above.

### T Cell Activation Assays.

Resting CD4+ T cells were treated with 20 ng/mL IL-7 (Miltenyi Biotec) and 10 IU/mL IL-2 (CFAR) in complete RPMI for 4 d. Cells were then infected by spinoculation as described above and cultured for 48 h in medium containing IL-7 and IL-2. After 48 h, the cells were activated by plate-bound anti-CD3 and soluble anti-CD28 in IL-2 supplemented RPMI medium. Cells were harvested 24 h postactivation and analyzed by flow cytometry.

### Statistical Analysis.

Statistical significance was calculated either using paired or unpaired Student’s *t* test or one-way ANOVA, and significance was assumed when *P* < 0.05. All statistical analyses were calculated using Prism 6 (GraphPad Prism).

### Data Availability Statement.

All data generated in this study are included in the paper and *SI Appendix*.

## Supplementary Material

Supplementary File
