# Supplementary material for: Loss of Nef-mediated CD3 down-regulation in the HIV-1 lineage increases viral infectivity and spread
Source: Proc Natl Acad Sci U S A. 2020 Mar 16;117(13):7382–91. doi: 10.1073/pnas.1921135117 (PMC7132320; doi:10.1073/pnas.1921135117)
Supplement: Supplementary File [file pnas.1921135117.sapp.pdf]

PNAS
www.pnas.org

Supplementary Information for

Loss of Nef-mediated CD3 downregulation in the HIV-1 lineage increases viral infectivity and spread

Dejan Mesner, Dominik Hotter, Frank Kirchhoff and Clare Jolly

Dr Clare Jolly

Email: c.jolly@ucl.ac.uk

This PDF file includes:

Supplementary materials
Figures S1 to S9

www.pnas.org/cgi/doi/10.1073/pnas.1921135117

Supplementary materials

Antibodies used for flow cytometry:

Primary antibodies: anti-HIV-1 p24-PE (KC57-RD1, Beckman Coulter); anti-HIV-1 Env clone PG9 (gift from Laura McCoy, UCL); anti-HIV-1 Env clone PGT151 (gift from Laura McCoy, UCL); anti-HIV-1 Env clone 50-69 (CFAR); anti-CD3-BV711 (UCHT1, Biolegend); anti-CD4-BV605 (SK3, Biolegend); anti-CD28-APC/R700 (CD28.2, BD); anti-CD69-APC/Fire780 (FN50, Biolegend); anti-phospho-S6-PE (pS235/pS236) (N7-548, BD); anti-CD38-PerCP/Cy5.5 (HIT2, Biolegend); anti-PD-1-PE/Cy7 (EH12.2H7, Biolegend); anti-CD247-PE (6B10.2, Biolegend); anti-HA.11 epitope tag-PE (16B12, Biolegend); anti-DYKDDDDK Flag tag-PE/Cy7 (L5, Biolegend). Secondary antibodies: anti-Human IgG-Cy5 (polyclonal,Bethyl); anti-Human IgG-PE (polyclonal, Jackson ImmunoResearch).

Antibodies for microscopy:

Primary antibodies: anti-HIV-1 Gag p17 and p24 rabbit antisera (donated by DR G. Reid and obtained from the CFAR); anti-HIV-1 gp41 Mab 50-69 (donated by Dr S. Zoller-Pazner and Dr M. Gorny, obtained from the CFAR). Secondary antibodies: anti-rabbit IgG-Cy3 (polyclonal, Jackson ImmnunoResearch); anti-human IgG-Cy5 (polyclonal, Bethyl).

Antibodies for western blotting:

Primary antibodies: anti-HIV-1 gp120 rabbit antisera (donated by Dr S. Ranjibar and obtained from the CFAR); anti-HIV-1 gp41 Mab 246-D (donated by Dr S. Zoller-Pazner and Dr M. Gorny, obtained from the CFAR); anti-HIV-1 Gag rabbit antisera (donated by DR G. Reid and obtained from the CFAR) and anti-tubulin (Sigma). Secondary antibodies: anti-Rabbit IgG (ab216773, Abcam), anti-Mouse IgG (ab216775, Abcam) and anti-Human IgG (926-32232, Licor).

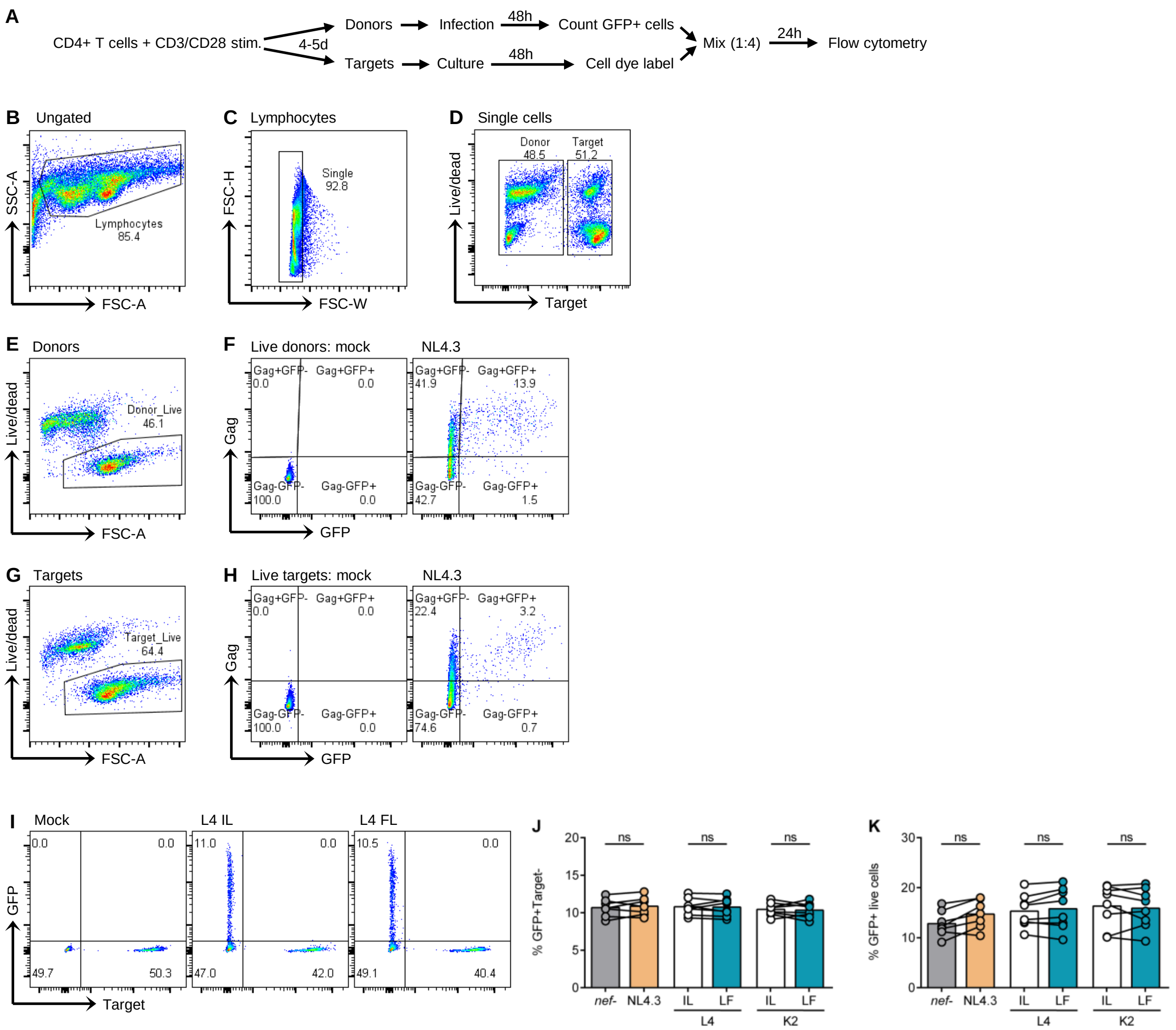

Fig. S1. Schematic of the cell-cell spread assay and gating strategy to analyze flow cytometry data. A) Schematic of the cell-cell spread assay. CD4+ T cells were stimulated with anti-CD3/CD28 antibodies for 4-5 days. Donor cells were infected by spinoculation and analyzed by flow cytometry to quantify GFP+ donor cells. Target cells (uninfected) were labeled with cell tracing dye (CPDe450) and mixed with infected donors cells in 1:4 ratio (GFP+ donor cells:target cells). Cells were analyzed by flow cytometry at 0h and 24h post-mixing to quantify cell-cell spread, expression of activation markers, and cell death. (B-H) Shown is a representative example from NL4.3 infected donor 24h post-mix. (B) Lymphocytes were gated based on their forward- and side-scatter properties (FSC-A vs SSC-A). (C) Doublets were excluded based on their forward scatter properties (FCS-W vs FSC-H). (D) Donor cells were gated as target dye (CPDe450) negative cells and target cells were gated as target dye positive cells. (E) Live donor cells were gated as Live/dead dye (Zombie UV) negative. (F) Infected live donor cells were identified as Gag+ or GFP+. Mock infected donor cells were used to set the gates. (G) Live target cells were gated as Live/dead dye negative. (H) Infected live target cells were identified as Gag+ or GFP+. Mock infected target cells were used to set the gates. (I-J) Flow cytometry analysis of donor-target cell co-culture at 0h post-mix. (I) Representative flow cytometry plots from indicated viruses. (J) Flow cytometry analysis showing equivalent infection levels were achieved in donor CD4+ T cells, quantified as percentage GFP+ve Target dye-ve cells (GFP+ donor cells) of total live cells. (K) Flow cytometry analysis of donor cells at 48h post-infection measuring percentage of GFP+ live cells. Bars show mean and lines join paired results from the same PBMC donor. Groups were compared using two-tailed paired *t*-test (ns, P>0.05; *, P<0.05; **, P<0.01).

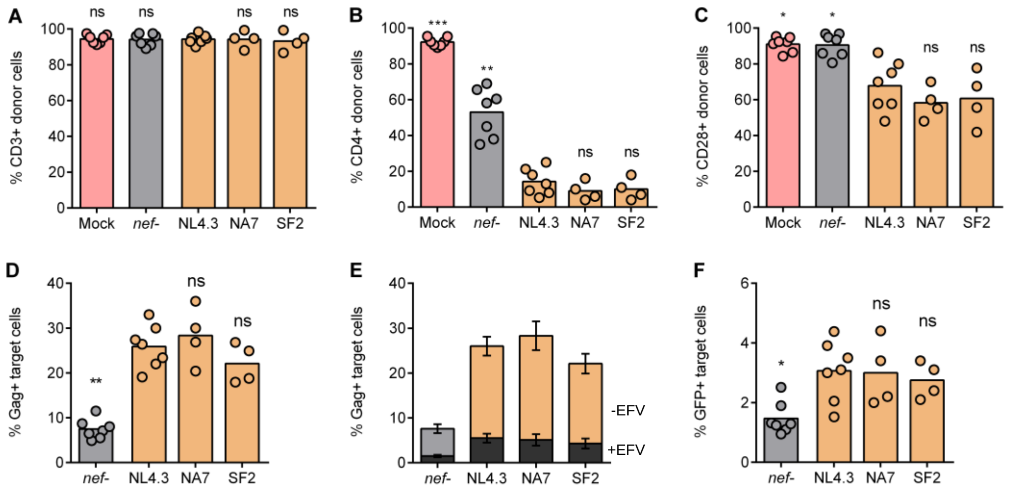

Fig. S2. Primary HIV-1 *nef* alleles have similar downmodulation and cell-cell spread properties compared to NL4.3 *nef*

Related to Figure 1. (A-C) CD4+ T cells were infected with indicated viruses and analyzed by flow cytometry 48h post-infection to measure expression of (A) CD3, (B) CD4, and (C) CD28 surface markers in infected Gag+GFP+ population. Total donor live population was analyzed for mock control. (D-F) CD4+ T cells were infected with indicated viruses for 48h and mixed with autologous pre-labelled target cells in 1:4 ratio. Cells were analyzed 24h post-mix by flow cytometry. (D) Percentage of Gag+ target cells. (E) Cells were cultured in presence or absence of 5 μM Efavirenz (± EFV). Grey bars are superimposed and show percentage of Gag+ targets in presence of Efavirenz (+EFV). (F) Percentage of GFP+ target cells. Bars show mean and symbols show individual PBMC donors. Error bars show mean ± SEM. All groups were compared against NL4.3 using one-way ANOVA with Sidak's multiple comparisons test (ns, P>0.05; *, P<0.05; **, P<0.01; ***, P<0.001).

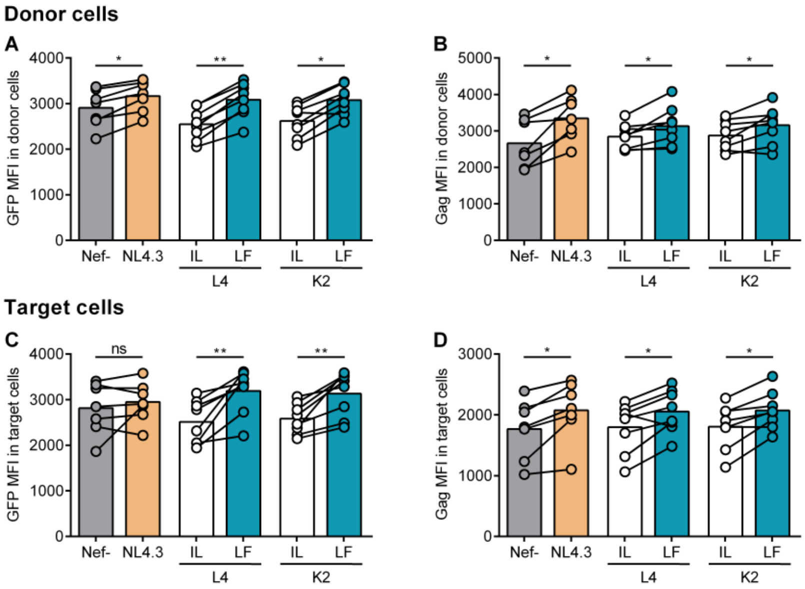

Fig. S3. Retained CD3 on infected cells results in increased Gag and GFP expression

Related to Figure 1 and 2. Primary CD4+ T cells were infected with indicated viruses for 48h, mixed with autologous pre-labelled target cells ratio and analyzed 24h post-mix by flow cytometry (n=8). (A) GFP expression (mean fluorescence intensity, MFI) in infected GFP+ donor cell population. (B) Gag expression in infected Gag+ donor cell population. (C) GFP expression in newly-infected GFP+ target cell population. (D) Gag expression in newly-infected Gag+ target cell population. Bars show mean and lines join paired results from the same PBMC donor. Groups were compared using two-tailed paired *t*-test (ns, P>0.05; *, P<0.05; **, P<0.01).

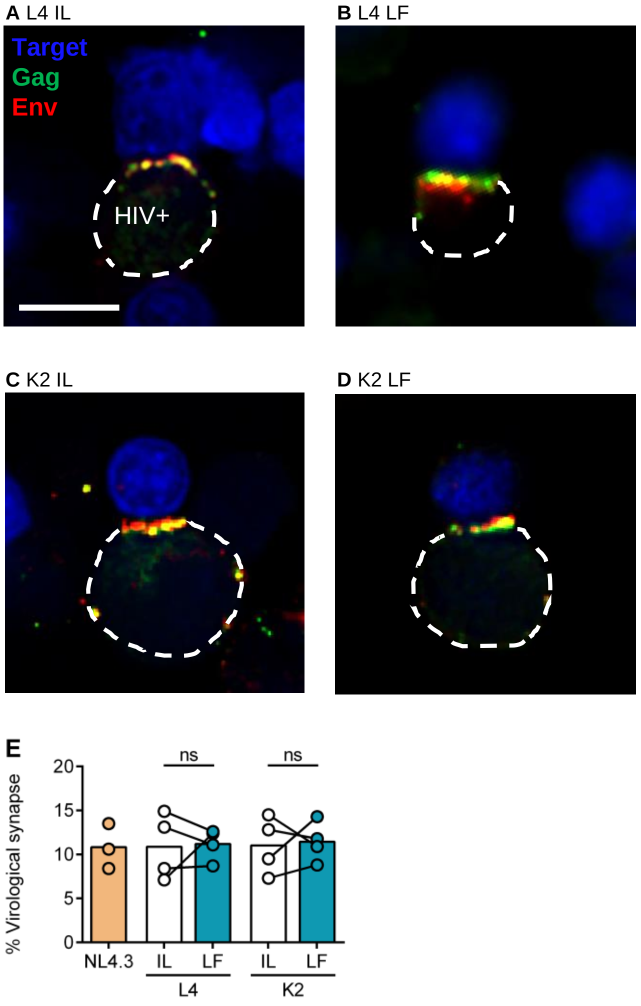

Fig. S4. CD4+ T cells infected with Nef chimeric viruses form virological synapses at similar frequencies

CD4+ T cells were infected with Nef chimeric viruses for 48h, mixed with autologous dye-labeled target cells and incubated on coverslips for 1h before fixation, staining and immunofluorescence microscopy. Virological synapse (VS) were defined as infected donor cell-target cell conjugate with Gag and Env polarized towards the cell-cell contact. (A-D) Target cell dye (blue), Gag (green), Env (red), and donor cell outline (white, dashed line). Scale bar is 5μm. Representative images (single *xy* slice) of VS for (A) L4 IL, (B), L4 LF, (C) K2 IL, and (D) K2 LF Nef chimeric viruses. (E) Percentage of donor-target cell conjugates forming VS. Minimum of 30 VS were counted for each PBMC donor. Bars show mean and lines join paired results from the same PBMC donor. Groups were compared using two-tailed paired *t*-test (ns, P>0.05; *, P<0.05).

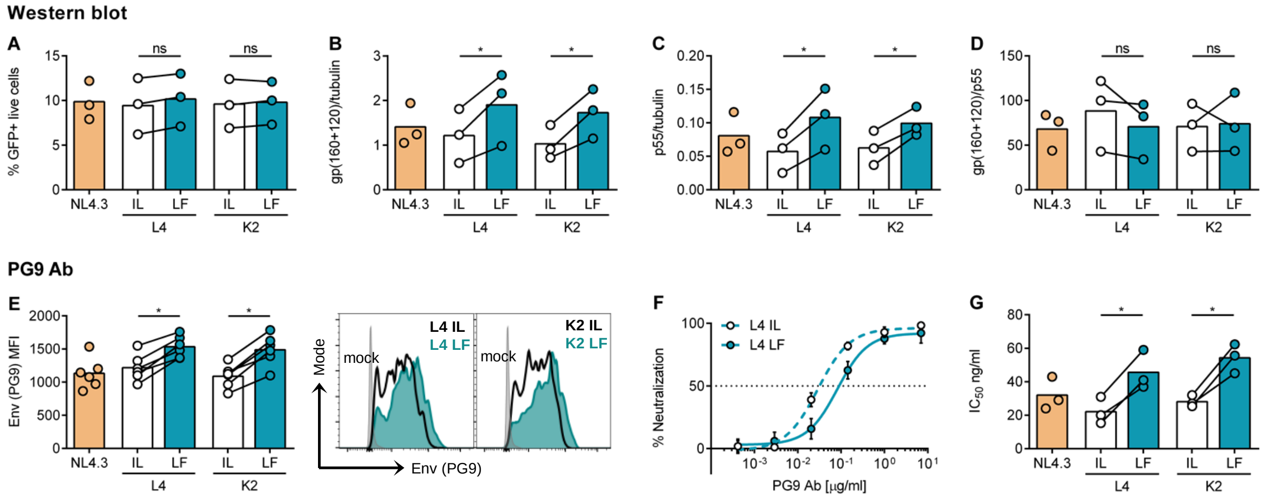

Fig. S5. Retained CD3 expression on infected cells increases Env levels in infected cells
Related to Fig. 3. (A-D) Quantification of western blotting of infected primary CD4+ T cell lysates from Fig. 3F (n=3). (A) Flow cytometry analysis of infected cells to measure percentage of GFP+ cells prior to cell lysis for western blotting. (B) Quantification of Env expression (gp160+gp120) normalized to tubulin. (C) Quantification of Gag expression (p55) normalized to tubulin. (D) Relative Env expression (gp160+gp120) normalized to p55. (E) Flow cytometry analysis of infected CD4+ T cells measuring cell surface expression of Env trimers as detected by PG9 Ab (n=6). (F-G) Neutralization assays were performed using virus containing supernatants from infected CD4+ T cells and PG9 Ab (n=3). (F) Representative examples of neutralization curves. (G) IC_{50} values calculated by non-linear regression analysis of the neutralization curves. Bars show mean and lines join paired results from the same PBMC donor. Error bars show mean ± SD. Groups were compared using two-tailed paired *t*-test (ns, P>0.05; *, P<0.05; **, P<0.01).

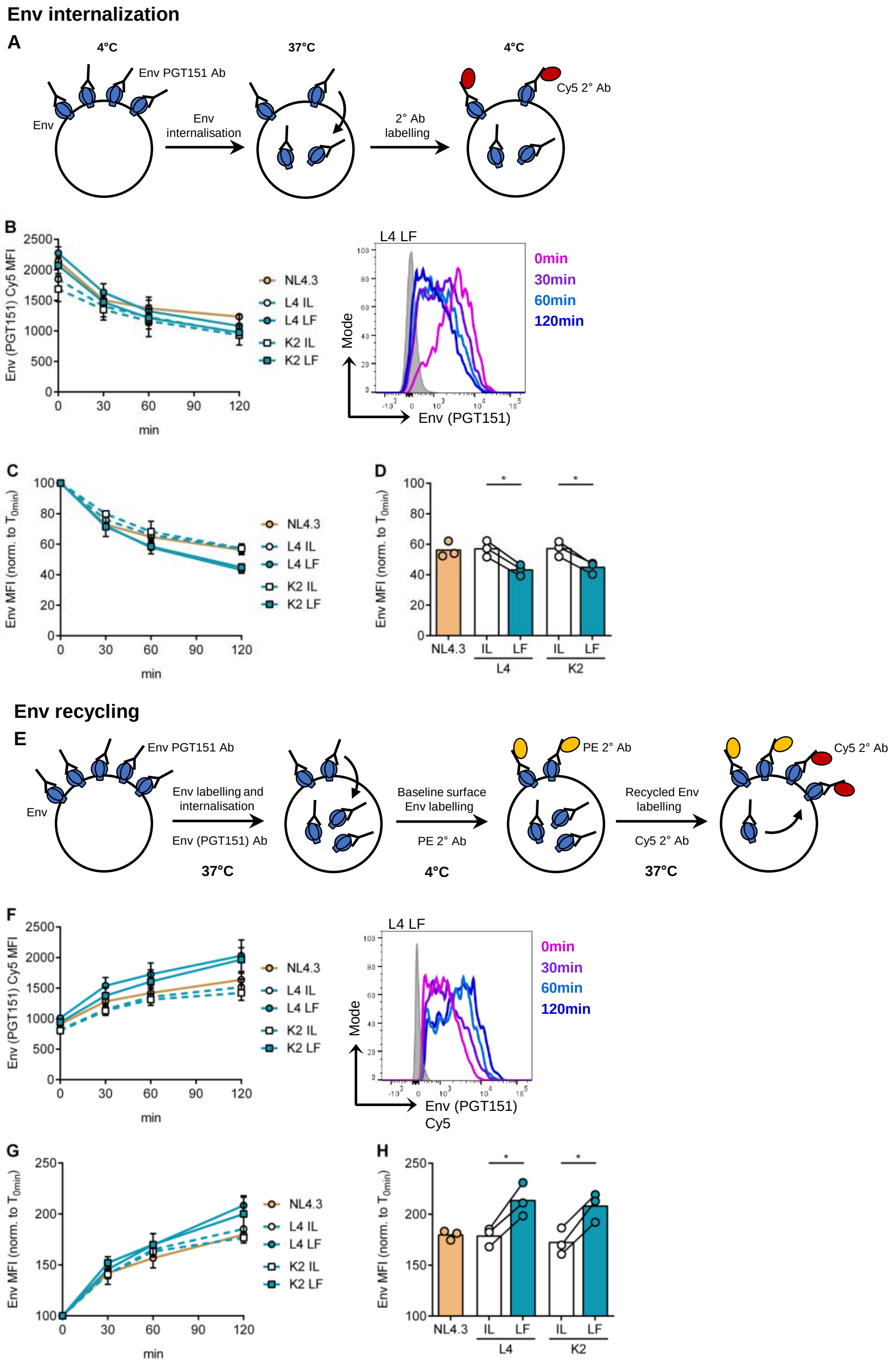

Fig. S6. Kinetics of Env internalization and recycling by Nef chimeric viruses

(A-D) Env internalization assay. (A) Schematic of the assay. (B) Quantification of remaining surface expression of Env (MFI) on primary CD4+ infected GFP+ cells after incubation at 37°C for the indicated time periods. Right panel shows a representative histogram for L4 LF Nef virus. (C) Env MFI values from (B) normalized to T_{0min}. The baseline (T_{0min}) Env MFI for each virus set as 100% and data show the percentage signal relative to 100% for each time point. (D) Normalized Env MFI at 120min. (E-H) Env recycling assay. (E) Schematic of the assay. (F) Surface expression of Env (MFI) as detected by Cy5-labelled secondary antibody on primary CD4+ infected GFP+ cells after incubation at 37°C for the indicated time periods. Right panel shows a representative histogram for L4 LF Nef virus. (G) Env MFI values from (F) normalized to T_{0min} as shown in (C). (H) Normalized Env MFI at 120min. Error bars show mean ± SEM. Bars show mean and lines join paired results from the same PBMC donor. Groups were compared using two-tailed paired *t*-test (ns, P>0.05; *, P<0.05; **, P<0.01; ***, P<0.001).

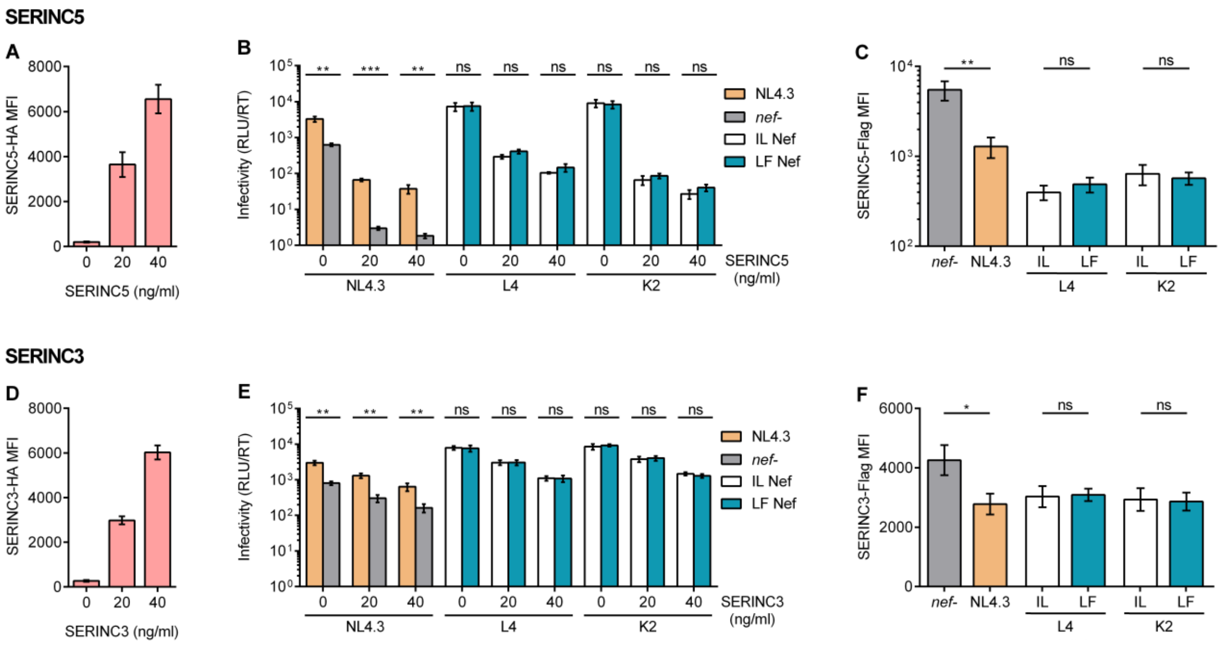

Fig. S7. Nef mutant viruses have similar ability to antagonize SERINC5/3

HEK293T cells were co-transfected with indicated proviruses and increasing doses of SERINC5 or SERINC3 plasmid. Cells and culture supernatants were harvested 48h post-transfection (n=3). Virion infectivity was measured as in Fig.3. SERINC expression was measured by flow cytometry, detecting the intracellular HA-tag or the extracellular Flag-tag. (A) Total SERINC5 expression (HA-tag) in transfected cells in the absence of virus. (B) Virion infectivity in the presence of SERINC5. (C) Cell surface expression of SERINC5 (Flag-tag) in cells co-transfected with the indicated viral plasmid and 20 ng/ml SERINC5. (D) Total SERINC3 expression (HA-tag) in cells transfected in the absence of virus. (E) Virion infectivity in the presence of SERINC3. (F) Cell surface expression of SERINC3 (Flag-tag) in cells transfected with the indicated viral plasmid and 20 ng/ml SERINC3. Error bars show mean ± SEM. Groups were compared using two-tailed unpaired *t*-test (ns, $P>0.05$; *, $P<0.05$; **, $P<0.01$; ***, $P<0.001$).

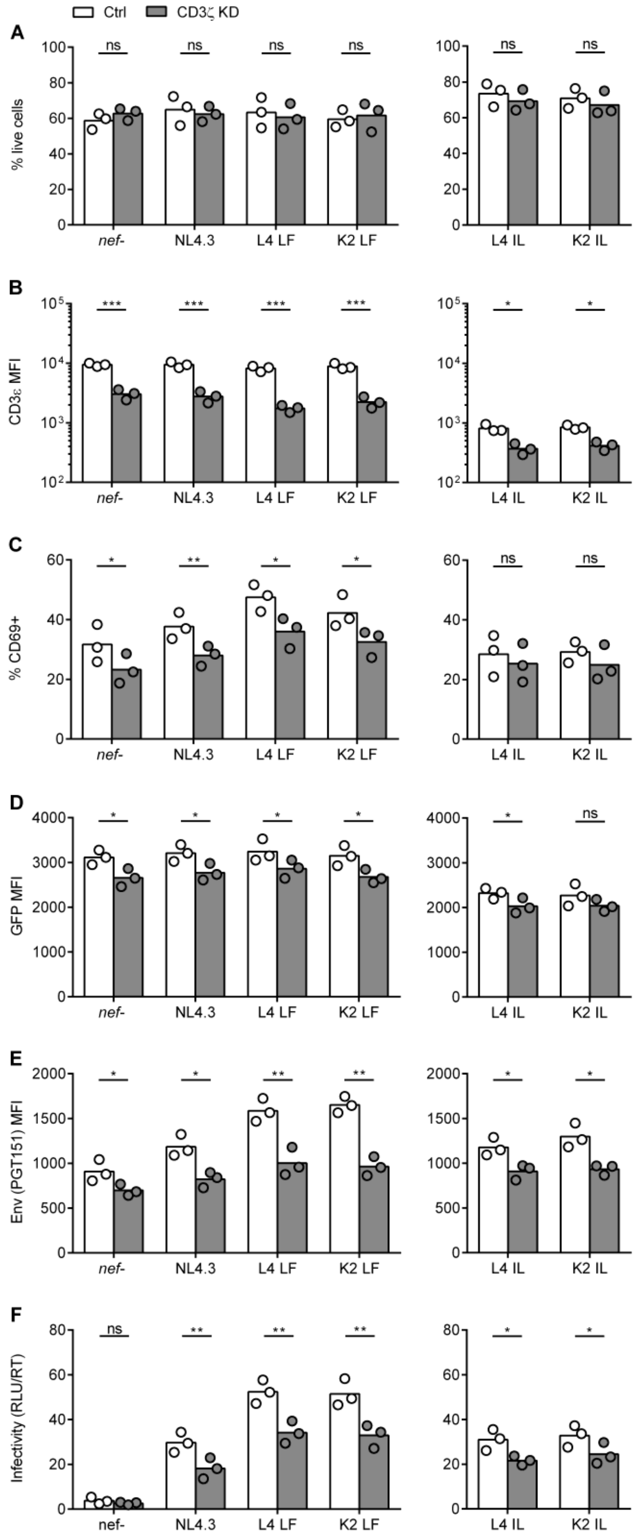

Fig. S8. Effects of CD3 RNAi knock-down on cells infected with viruses that do and do not downmodulate CD3. Related to Fig. 4. Activated CD4 T cells were electroporated with siRNA to knock-down CD3ζ (CD247) or a non-targeting control for 48h and infected with indicated viruses (n=3). (A-E) 48h post-infection cells were analyzed by flow cytometry to measure (A) cell viability and expression of (B) CD3ε, (C) CD69, (D) GFP, and (E) Env trimers (PGT151) on GFP+ cells. (F) Virus supernatant was collected at 48h post-infection and analyzed for virion infectivity as described in Fig.3. Bars show mean and symbols show individual PBMC donors. Groups were compared using two-tailed paired *t*-test (ns, P>0.05; *, P<0.05; **, P<0.01; ***, P<0.001).

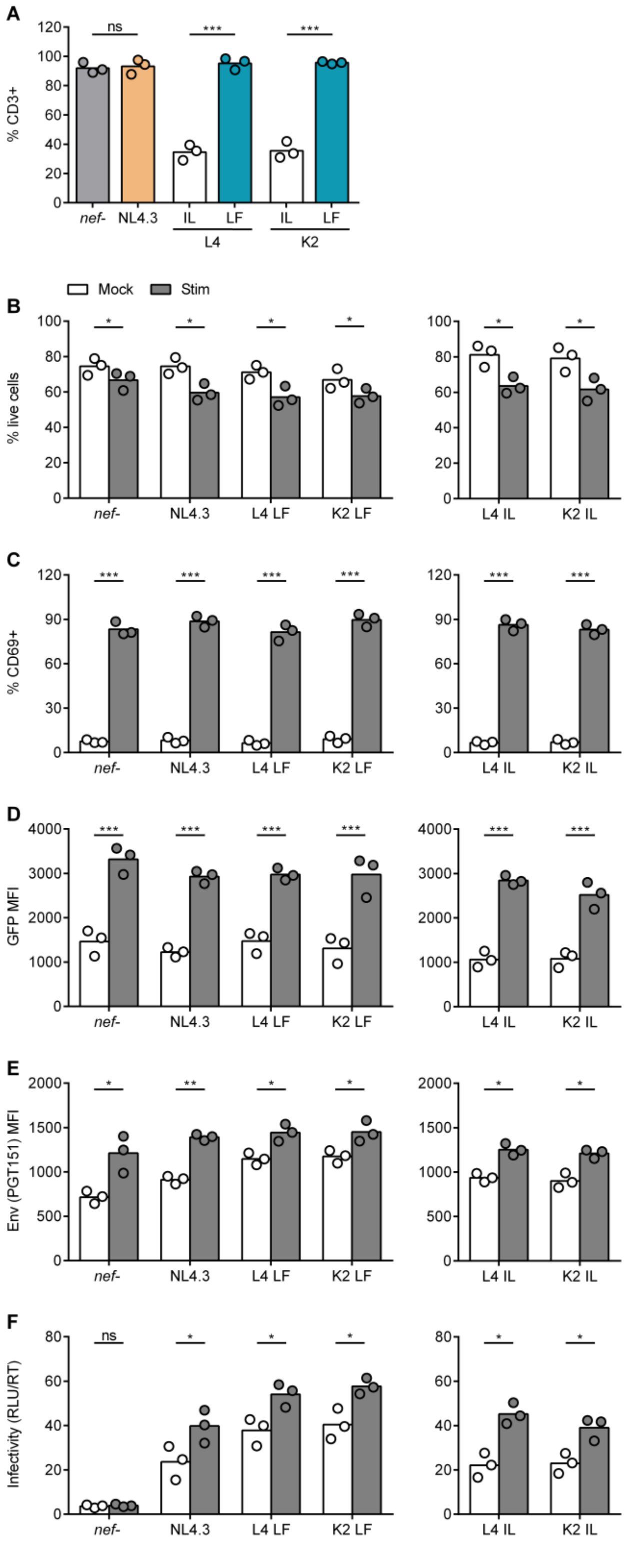

Fig. S9. Env expression and virion infectivity is increased by T cell activation

Related to Fig. 5. Resting CD4+ T cells were treated with IL-7 for 4 days and then infected with indicated viruses. (A) Infected cells were analyzed by flow cytometry 48h post-infection (prior to stimulation) to measure percentage of CD3+ cells in GFP+ population. 48h post-infection cells were either stimulated with anti-CD3/CD28 antibodies or unstimulated (mock) for 24h and analyzed by flow cytometry to measure (B) cell viability and expression of (C) CD69 in total live population, (D) GFP in GFP+ population, and (G) Env trimers (PGT151) in GFP+ population. (F) Virus supernatant was collected 24h post-stimulation and analyzed for virion infectivity as described in Fig.3. Bars show mean and symbols show individual PBMC donors. Groups were compared using two-tailed paired *t*-test (ns, P>0.05; *, P<0.05; **, P<0.01; ***, P<0.001).
